# Predictive performance of comorbidity measures in administrative databases for diabetes cohorts

**DOI:** 10.1186/1472-6963-13-340

**Published:** 2013-09-02

**Authors:** Lisa M Lix, Jacqueline Quail, Opeyemi Fadahunsi, Gary F Teare

**Affiliations:** 1 Department of Community Health Sciences, University of Manitoba, Winnipeg, MB, Canada; 2 School of Public Health, University of Saskatchewan, Saskatoon, SK, Canada; 3 Saskatchewan Health Quality Council, Saskatoon, SK, Canada; 4 Department of Community Health and Epidemiology, University of Saskatchewan, Saskatoon, SK, Canada; 5 Department of Medicine, Reading Hospital, West Reading, PA, USA

**Keywords:** Administrative health data, Comorbid conditions, Diabetes, Risk adjustment, Statistical models

## Abstract

**Background:**

The performance of comorbidity measures for predicting mortality in chronic disease populations and using ICD-9 diagnosis codes in administrative health data has been investigated in several studies, but less is known about predictive performance with ICD-10 data and for other health outcomes. This study investigated predictive performance of five comorbidity measures for population-based diabetes cohorts in administrative data. The objectives were to evaluate performance for: (a) disease-specific and general health outcomes, (b) data based on the ICD-9 and ICD-10 diagnoses, and (c) different age groups.

**Methods:**

Performance was investigated for heart attack, stroke, amputation, renal disease, hospitalization, and death in all-age and age-specific cohorts. Hospital records, physician billing claims, and prescription drug records from one Canadian province were used to identify diabetes cohorts and measure comorbidity. The data were analysed using multiple logistic regression models and summarized using measures of discrimination, accuracy, and fit.

**Results:**

In Cohort 1 (*n* = 29,058), for which only ICD-9 diagnoses were recorded in administrative data, the Elixhauser index showed good or excellent prediction for amputation, renal disease, and death and performed better than the Charlson index. Number of diagnoses was a good predictor of hospitalization. Similar results were obtained for Cohort 2 (*n* = 41,925), in which both ICD-9 and ICD-10 diagnoses were recorded in administrative data, although predictive performance was sometimes higher. For age-specific models of mortality, the Elixhauser index resulted in the largest improvement in predictive performance in all but the youngest age group.

**Conclusions:**

Cohort age and the health outcome under investigation, but not the diagnosis coding system, may influence the predictive performance of comorbidity measure for studies about diabetes populations using administrative health data.

## Background

Administrative health data are frequently used for surveillance and research in chronic disease populations. These data contain medical records generated for management and remuneration purposes at the time of hospital discharge or provision of services [1]. Besides providing timely and cost-effective information, their popularity stems from the fact that they are population-based and capture both utilization and diagnostic information. However, to obtain unbiased conclusions from observational chronic disease studies using administrative data requires control of confounding factors that may differ among populations and are associated with the health utilization or outcome measure under investigation. Demographic and socioeconomic variables are included as risk-adjustment measures in most observational studies. Comorbid conditions, pre-existing conditions that co-occur with the index disease, [2] are also commonly considered.

A number of comorbidity measures are available for administrative health data. These include both general-purpose and disease-specific comorbidity measures; [3,4] general-purpose measures are advantageous because they can be used to compare comorbidity characteristics across different chronic disease populations. Some general measures are based on simple counts of the number of diagnoses or prescription drugs for an individual [5]. Others are based on specific sets of diagnosis codes or prescription drug codes. The Chronic Disease Score (CDS), for example, is based on a set of codes for prescription drugs used to treat major chronic conditions [6]. Diagnosis-related measures, such as the Charlson and Elixhauser indices, use International Classification of Disease (ICD) diagnosis codes to identify major comorbid conditions [7,8]. Both the Charlson and Elixhauser indices were originally used to predict mortality for in-hospital populations, but they have also been applied to outpatient populations and to some other health outcomes [9-11]. The Elixhauser index was developed using the clinical modification of the 9^th^ revision of ICD (i.e., ICD-9-CM); the Charlson index was also proposed using this classification system. However, many countries, including Canada, Australia, New Zealand, Japan, China, and some European countries have now implemented the 10^th^ revision of ICD (i.e., ICD-10), which covers a broader range of clinical information. Quan et al. [12] extended the Charlson and Elixhauser indices to ICD-10 codes, but only a few studies have compared the predictive performance of comorbidity algorithms based on ICD-9 and ICD-10 codes. Li et al. [13] observed good predictive performance for in-hospital mortality using both the Charlson and Elixhauser indices with the two coding systems in Canadian data. Sundararajan et al. [14] found similar results using Australian data and when the outcome was in-hospital mortality. However, the authors noted that predictive performance for other outcomes could be investigated.

Several studies have used administrative data to study health outcomes and healthcare use in diabetes populations. Diabetes places a significant burden on the health care system [15-18], and therefore is of great interest to clinicians and policy analysts. It is responsible for vascular and neurologic complications such as acute myocardial infarction (AMI), stroke, lower extremity amputation (LEA), end stage renal disease (ESRD), and retinopathy [15-20]. De Berardis et al. found that the hospitalization rate in diabetics is twice that of the general population, accounting for an excess of 12,000 hospital admissions per 100,000 person years [15]. Studies that have investigated comorbidity measures in diabetes populations using administrative data have been limited, although Quail et al. [21] found that the predictive performance of different comorbidity measures for mortality and hospitalization outcomes was variable in study cohorts with diabetes.

Only a few studies have compared the predictive performance of comorbidity measures in different age groups, although these groups may differ in their comorbidity characteristics. Studies that have investigated risk-adjustment tools have often focused on older populations [3,22,23]. In contrast, Quail et al. compared an age-inclusive cohort (i.e., 20+ years) to an age-restricted cohort (i.e., 65+ years) and found diminished performance of comorbidity measures in predicting mortality and hospitalization in the latter [21].

Given this background, the study purpose was to investigate predictive performance of comorbidity measures in diabetes cohorts defined from administrative health data. The objectives were to compare performance for: (a) disease-specific and general health outcomes, (b) data based on ICD-9 and ICD-10 diagnoses, and (c) different age groups.

## Methods

### Data sources

Study data were from the Canadian province of Saskatchewan, which has a population of approximately 1.1 million according to the 2006 national census. About 40 per cent of residents live in one of two major urban centres, while the remainder of residents live in rural communities [24].

Like other Canadian provinces and territories, Saskatchewan has a universal health care system. All hospital records and virtually all physician billing claims and outpatient prescription drug records are captured for residents eligible to receive health insurance benefits. Records of hospital, physician, and prescription drug services are collected in electronic databases that can be anonymously linked, via a unique personal health number, to the population health insurance registration file [25]. The registration file captures dates of health insurance coverage, demographic characteristics and location of residence.

A hospital record is completed upon patient discharge. Diagnoses are recorded using ICD-9 up to and including the 2000/01 fiscal year (a fiscal year extends from April 1 to March 31). Beginning in 2001/02, ICD-10-CA diagnoses were used. Between three and 16 diagnoses are captured in the data prior to the introduction of ICD-10-CA and up to 25 diagnoses are captured subsequently.

Physicians paid on a fee-for-service basis submit billing claims to the ministry of health for payment purposes. A single diagnosis is recorded on each claim using three-digit ICD-9 codes.

Prescription drug files contain records of outpatient drugs dispensed to residents eligible for coverage. Registered Indians, who represent about 9 per cent of the population, have their prescription drug benefits paid by the federal government rather than the province so their records are not available in the provincial database. Prescription drug records include the date of dispensation and national drug identification number (DIN). DINs are linked to codes in the American Hospital Formulary System (AHFS) Pharmacologic-Therapeutic Classification System (http://www.ashp.org). The AHFS is used to group drugs with similar pharmacologic, therapeutic, and/or chemical characteristics using a hierarchical system with four levels.

The accuracy and completeness of Saskatchewan’s administrative data for research has been documented in multiple studies [26-28]. Ethics approval for this research was received from the University of Saskatchewan Biomedical Research Ethics Board. Data were accessed and analysed at the Health Quality Council in accordance with a standing data sharing agreement between that organization and the provincial health ministry.

### Study cohorts

To permit comparisons of ICD coding systems, two cohorts were defined using the diabetes case definition developed by the National Diabetes Surveillance System, [29] which has been validated in previous research [30,31]. This case definition identifies all individuals with a diabetes diagnosis (ICD-9 250; ICD-10-CA E10-E14) in at least one hospital record or two physician claims within a two-year period. The diagnosis index date is the date of hospitalization or the date of the last of the two physician visits.

Cohort 1 was composed of residents aged 20 years and over at their diagnosis index date who satisfied the diabetes case definition and had uninterrupted health coverage from 1997/98 to 1999/00 or until death. The ICD-9 coding system was exclusively used during this period to record diagnoses in both hospital and physician databases. Cohort 2 was composed of residents aged 20 years and over at their diabetes index date who satisfied the diabetes case definition and had uninterrupted health coverage from 2001/02 to 2003/04 or until death. ICD-9 (for physician data) and ICD-10 (for hospital data) coding systems were in use during this latter period. Data from fiscal year 1996/97 onward, the first year of available data, were used to ascertain diabetes cases.

### Comorbidity measures

Five measures were investigated (Table 1): number of different diagnoses, Charlson index, Elixhauser index, number of different drugs, and CDS. For Cohort 1, data from 1997/98 were used to define each measure while for Cohort 2, data were from 2001/02.

**Table 1:** Description of comorbidity measures

**Measure**	**Definition**	**Data Source**	**Variable Categories for the Predictive Model**^**a**^
Number of diagnoses	Identifies number of diagnosis codes, to the third digit. Minimum score is 0 and there is no maximum	Hospital records and physician billing claims	0−2 (reference), 3–5, 6–9, 10 or more
Charlson index	Summarizes diagnoses for 17 conditions. Total score can range from 0 to 32	Hospital records and physician billing claims	0−1 (reference), 2–3, 4–5, 6 or more
Elixhauser index	Captures diagnoses for 31 conditions. No total score is produced	Hospital records and physician billing claims	Dummy variables (0 = absent; 1 = present) are used to define the 31 conditions
Number of drugs	Identifies number of prescription drugs using six-digit AHFS codes. Total score can range from 0 to 125	Prescription drug records	0−1 (reference), 2–3, 4–5, 6 or more
Chronic Disease Score (CDS)	Uses AHFS codes to identify number of different prescriptions for treating 17 conditions. Total score can range from 0 to 35	Prescription drug records	0−1 (reference), 2–3, 4–5, 6–7, 8 or more

^a^Variable categories were defined based on previous research and by examining the frequency distributions for each of the comorbidity measures. Cut-points for the categories were created to minimize sparse cell sizes, *AHFS* American Hospital Formulary System.

The number of different diagnoses in hospital and physician data [32] was based on codes recorded to the third digit in both ICD-9 (e.g., 812) and ICD-10 (e.g., S42). Diagnoses related to pregnancy, childbirth, or abortions were excluded. The Charlson index was originally developed using data abstracted from hospital charts, [7] subsequently adapted to hospital data coded with ICD-9, and then extended to data coded with ICD-10 [12]. The index is based on diagnoses for 17 conditions; each condition is assigned a weight from one to six. A summary score is computed ranging from 0 to 32, where a higher score indicates greater comorbidity. In accordance with previous research, [33] the Charlson index was computed using diagnosis codes in both hospital and physician data. For hospital data, only those conditions present on admission were included. The Elixhauser index [8] has also been extended from its original formulation using ICD-9 to ICD-10 [12]. Each of the 31 conditions comprising the index is coded as a binary indicator. This index was also implemented using diagnosis codes from both hospital and physician data. The number of different prescription drugs has also been shown to be a valid measure of comorbidity [11]. This measure was based on six-digit AHFS codes, of which there are 125. The CDS [6] was originally developed for predicting mortality and hospitalization outcomes. It uses the first four digits of AHFS codes to identify prescription drugs for treating 17 conditions. Each disease treatment group is assigned a weight and the weighted category scores are used to produce a single summary score from 0 to 35, where a high score indicates greater comorbidity.

### Outcome variables

Study outcomes were identified using data from the two years following the comorbidity measurement year of each cohort. Thus, outcomes were measured for 1998/99 to 1999/00 for Cohort 1 and 2002/03 to 2003/04 for Cohort 2. Outcomes of AMI, stroke, LEA, and ESRD were based on previously-defined algorithms [34-36]. Specifically, AMI cases were identified with an ICD-9 code of 410 or ICD-10 code of I21 in the most responsible (i.e., primary) diagnosis field in hospital records. AMI hospitalizations that occurred within a one-year period following a previous AMI were excluded, to ensure that only incident cases were captured. Stroke cases were identified using ICD-9 codes 430, 431, 434, 436, and 362.3 and ICD-10 codes I60, I61, I63, I64, and H34.1 in the most responsible diagnosis field in hospital records. The LEA case definition was based on procedure codes in hospital records. It captured both minor (toes, forefoot, foot below ankle), and major (ankle, below knee, above knee) amputation procedures not related to trauma or malignancy. Individuals with ESRD were identified from service codes for chronic dialysis and renal transplantation. The full list of procedure and service codes for LEA and ESRD is available from the authors.

Other outcomes that were investigated included death, hospitalization for any reason, and hospitalization for diabetes (ICD-9 250; ICD-10-CA E10-E14). Deaths were identified from the population registry. Hospitalizations associated with pregnancy, childbirth, or abortions were excluded. Transfers between facilities and hospital re-admissions within 24 hours of discharge were considered part of the initial hospital admission [35].

### Other study variables

The cohorts were described on age, sex, recent diabetes diagnosis, region of residence, and income quintile. Age and sex were defined from the population registration file. Individuals who had a diagnosis index date in 1997/98 or 2001/02 for Cohorts 1 and 2, respectively, were identified as recently diagnosed. Individuals with a prior index date were defined as previously diagnosed. Urban and rural region of residence was defined based on postal code in the registration file; urban residents were those living in the health regions of Saskatoon and Regina.

Income quintile was defined using a method based on average household income from the 2001 Statistics Canada Census [37]. Each individual’s postal code was assigned to a dissemination area (DA), the smallest geographic unit for which Census data are reported. Income ranges were determined such that the entire Saskatchewan population was divided into five approximately equal groups. Residents were assigned an income quintile according to their DA average household income. Some residents could not be assigned a quintile because income measures are suppressed for some DAs, usually because of small population size. Approximately 14 per cent of the total population had a missing income quintile. A method was used that first developed a predictive model for the missing quintiles based on socio-demographic variables that are generally not suppressed, including marital status, ethnicity, and unemployment. A multiple imputation approach was then used to assign income quintile, taking the average of the multiple imputed values, [38] for all DAs that did not have missing information on one or more of these socio-demographic variables and for all individuals who did not have a missing postal code. Average total income, reported by Statistics Canada in 2001, was $12,700 for the lowest quintile, $29,700 for the second quintile, $47,200 for the third quintile, $71,800 for the fourth quintile, and $128,700 for the highest quintile (http://www5.statcan.gc.ca/cansim).

### Statistical analysis

Frequencies, means, and standard deviations were used to describe the cohorts’ characteristics. Performance of each comorbidity measure was assessed using multiple logistic regression by fitting base and full models to the data [32]. For each cohort, the base model contained the following variables: age (centred on the mean), a quadratic age term, sex, region of residence (urban [reference], rural), income quintile (Q1/Q2, Q3 [reference], Q4/Q5), and recent diabetes diagnosis (prior [reference], recent). The full model contained all variables in the base model in addition to a comorbidity variable(s), which were modelled as categorical variables. The variable categorization adopted for each comorbidity measure is provided in Table 1.

The base and full models were compared using the *c*-statistic, a measure of discrimination that is equivalent to the area under the receiver operating characteristic curve for dichotomous outcomes [39,40]. The *c*-statistic ranges from zero to one, with a value of one representing perfect prediction and a value of 0.5 representing chance prediction. A value between 0.7 and 0.8 is considered to demonstrate acceptable predictive performance, while a value greater than 0.8 demonstrates excellent predictive performance. The 95% confidence intervals (CIs) were computed. The difference in *c*-statistics for the base and full models (i.e., Δ*c*) was tested for statistical significance using the method of DeLong et al. [41]. The percentage change in the *c*-statistic between base and full models was also computed.

To investigate model fit, a likelihood ratio test (LRT) was also conducted for the base and full models [42]. The LRT statistic asymptotically follows a χ^2^ distribution, with the degrees of freedom (df) for this statistic equal to the difference in df for the base and full models. A statistically significant LRT statistic indicates that the inclusion of the comorbidity measure results in an improvement in model fit. Each test was conducted at the *α* = .01 significance level, to reduce the overall probability of a Type I error.

The Brier score, which combines information about model calibration (i.e., accuracy) and discrimination, was also computed. The Brier score ranges from zero to one, [43] with lower values indicating less prediction error. Given that a score of 0.25 can be achieved by assigning an event probability of 0.5 to each individual, [43] a value less than 0.25 was considered to represent acceptable prediction error.

All analyses were conducted using SAS software [44]. Separate analyses were conducted for Cohorts 1 and 2 and for age-specific groups within each cohort. The age-specific groups were: 20 to 44 years, 45 to 64 years, 65 to 74 years and 75+ years.

## Results and discussion

Cohort 1 consisted of a total of 29,058 individuals and Cohort 2 consisted of 41,925 individuals. In total, 1,106 (3.7%) individuals were excluded from Cohort 1 because they did not have health insurance coverage throughout the study observation period; this percentage was similar for Cohort 2 (3.9%). Table 2 describes the age-specific demographic, health outcome, and comorbidity characteristics for both cohorts. The youngest age group was more likely to be comprised of urban residents while the oldest age group was more likely to contain rural residents. In Cohort 1, close to one-third of individuals in the youngest age group were in the lowest income quintile, compared to 27.1% of individuals in the 75+ age group. Similar results were observed for Cohort 2. Overall, individuals in Cohort 1 were more likely to have a recent diabetes diagnosis compared to Cohort 2. The overall percentage of individuals experiencing each health outcome was higher in Cohort 1 than Cohort 2 with the exception of ESRD. Cohort 1 had lower mean scores for the number of diagnoses, number of drugs and CDS, but not the Charlson index score, for which both cohorts had the same mean score. As expected, the average scores for the comorbidity measures increased with age.

**Table 2:** Description of diabetes cohorts

	**Cohort 1**^**a **^**(*n*=29,058)**					**Cohort 2 (*n*=41,925)**				
	**20-44 (n=3,901)**	**45-64 (n=10,659)**	**65-74 (n=7,757)**	**75+ (n=6,741)**	**All**	**20-44 (n=3,901)**	**45-64 (n=10,659)**	**65-74 (n=7,757)**	**75+ (n=6,741)**	**All**
**Demographics**^**b**^										
Age, mean (SD)	36.2 (6.3)	55.5 (5.6)	69.5 (2.8)	80.6 (4.6)	62.5 (15.0)	36.8 (5.8)	55.3 (5.6)	69.6 (2.9)	81.0 (4.8)	62.4 (15.0)
Female	52.1	44.1	46.0	53.5	47.9	52.3	43.9	44.9	54.5	47.8
Urban	58.3	53.0	48.7	46.3	51.0	55.6	53.4	51.1	47.8	51.8
Missing	<0.1	0.2	0.1	<0.1	0.1	0.1	0.2	0.1	<0.1	0.1
Income Quintile										
Q1 (lowest)	32.7	26.1	25.8	27.1	27.1	32.5	25.7	24.4	25.0	26.1
Q2	22.0	23.2	23.2	24.7	23.4	23.2	22.1	23.4	23.8	23.0
Q3	15.4	16.2	17.1	19.1	17.0	14.4	16.4	17.5	19.1	17.0
Q4	13.5	14.8	15.1	12.8	14.2	13.0	15.1	15.4	14.6	14.8
Q5 (highest)	15.4	18.2	17.4	15.1	16.9	16.0	19.5	18.0	16.4	17.9
Missing	0.9	1.5	1.5	1.1	1.3	1.0	1.3	1.4	1.2	1.2
Recent diagnosis	27.5	24.3	21.7	21.3	23.4	16.0	12.6	9.6	8.3	11.3
**Outcomes (%)**^c^										
AMI	0.5	1.6	2.9	3.3	2.2	0.4	0.9	1.9	3.0	1.6
Stroke	0.3	1.0	2.7	3.7	2.0	0.2	0.7	1.8	2.9	1.4
LEA	0.3	0.8	1.2	1.3	1.0	0.3	0.9	0.9	0.6	0.8
ESRD	0.7	0.5	0.4	0.2	0.4	0.3	0.5	0.5	0.3	0.4
Mortality	0.9	4.0	10.0	22.0	9.4	1.2	3.0	8.4	21.5	8.4
Hosp., any	34.3	43.7	57.6	64.7	51.0	31.7	40.9	55.2	61.8	48.1
Hosp., diabetes	18.6	21.3	26.6	30.2	24.4	14.5	16.8	22.0	24.0	19.5
**Comorbidity summary measures, mean (SD)**										
# diagnoses	6.0 (4.8)	6.2 (4.8)	6.7 (4.7)	7.4 (4.8)	6.6 (4.8)	6.2 (5.7)	6.8 (6.0)	7.9 (6.6)	8.9 (6.8)	7.5 (6.4)
Charlson score	0.4 (0.8)	0.6 (1.2)	0.9 (1.5)	1.2 (1.7)	0.8 (1.4)	0.4 (0.8)	0.6 (1.2)	1.0 (1.7)	1.3 (1.9)	0.8 (1.5)
# drugs	3.1 (3.2)	4.2 (3.5)	5.3 (3.6)	5.9 (3.8)	4.7 (3.7)	3.1 (3.3)	4.5 (3.7)	5.9 (3.8)	6.4 (4.0)	5.1 (3.9)
CDS	2.5 (3.1)	3.9 (3.5)	5.0 (3.6)	5.3 (3.6)	4.3 (3.6)	2.8 (3.4)	4.4 (3.8)	5.6 (3.7)	5.7 (3.7)	4.8 (3.8)

^a^Cohort 1 was defined using administrative data from fiscal years 1996/97 to 1997/98 and Cohort 2 was defined using data from 1996/97 to 2001/02; ^b^Numbers reported are percentages unless otherwise indicated; ^c^Outcomes were defined over a two-year observation period, *SD* Standard Deviation, *Hosp* Hospitalization, *AMI* Acute Myocardial Infarction; *LEA* Lower Extremity Amputation; *ESRD* End-Stage Renal Disease, *CDS* Chronic Disease Score.

Tables 3 and 4 describe the comorbidities comprising the Charlson and Elixhauser indices, respectively. For the Charlson index, the most common comorbidities in both cohorts were uncomplicated diabetes and chronic pulmonary disease. For the Elixhauser index, the most common comorbidities, in addition to uncomplicated diabetes, were uncomplicated hypertension and chronic pulmonary disease. More than 60% of individuals in both Cohorts 1 and 2 had at least one of the Elixhauser comorbidities.

**Table 3:** Charlson index comorbidities (%) for study cohorts

**Comorbidity**	**Cohort 1**^**a **^**(*n *= 29,058 )**	**Cohort 2 (*n *= 41,925)**
Diabetes, without complications	15.5	15.3
Chronic pulmonary disease	14.0	13.7
Congestive heart failure	8.0	8.1
Cancer	5.5	5.8
Cerebrovascular disease	5.5	4.7
Myocardial infarction	2.5	3.0
Diabetes, with complications	2.3	2.5
Peptic ulcer disease	2.0	1.7
Renal disease	1.9	3.2
Peripheral vascular disease	1.4	1.6
Metastatic carcinoma	1.3	1.8
Connective tissue disease	0.8	0.8
Dementia	0.8	0.9
Mild liver disease	0.6	0.6
Paraplegia and hemiplegia	0.6	0.4
Moderate or severe liver disease	0.1	0.2
AIDS/HIV	<0.1	<0.1
**1 or more Charlson comorbidities**	**39.1**	**37.2**

^a^Charlson index comorbidities were defined using administrative data from fiscal year 1997/98 for Cohort 1 and 2001/02 for Cohort 2.

**Table 4:** Elixhauser index comorbidities (%) for study cohorts

**Comorbidity**	**Cohort 1**^**a **^**(*n *= 29,058)**	**Cohort 2 (*n *= 41,925)**
Hypertension- uncomplicated	33.3	42.7
Diabetes- uncomplicated	14.7	14.7
Chronic pulmonary disease	13.9	13.5
Congestive heart failure	8.0	8.0
Depression	5.8	6.0
Solid tumors	5.0	5.4
Diabetes- complicated	3.2	3.0
Psychiatric	2.4	2.7
Cardiac arrhythmias	2.3	2.8
Fluid and electrolyte disorders	1.9	2.7
RA/collagen vascular disease	1.9	1.9
Renal failure	1.8	3.1
Valvular disease	1.5	2.2
Peripheral vascular disease	1.4	1.5
Metastatic cancer	1.3	1.7
Other neurological disorders	1.1	1.2
Coagulopathy	1.0	2.5
Hypertension- complicated	0.7	1.0
Pulmonary circulatory disorders	0.7	0.8
Liver disease	0.6	0.7
Obesity	0.6	1.0
Paraplegia	0.6	0.4
Deficiency anemia	0.5	0.8
Alcohol abuse	0.4	0.7
Hypothyroidism	0.4	1.1
Drug abuse	0.3	0.4
Peptic ulcer disease	0.3	0.4
Lymphoma	0.2	0.2
Weight loss	0.1	0.2
AIDS	<0.1	<0.1
Blood loss anemia	0.0	<0.1
**1 or more Elixhauser comorbidities**	**61.4**	**65.5**

^a^Comorbidities were defined using data from fiscal year 1997/98 for Cohort 1 and 2001/02 for Cohort 2.

Table 5 reports the modelling results for both cohorts when age-inclusive analyses were conducted. The LRTs were statistically significant for all comorbidity measures and for all outcomes, except for the CDS for AMI and the Elixhauser index for stroke in Cohort 2. These results indicate that the comorbidity measures almost always resulted in an improvement in model fit. Therefore, the focus of the remainder of this section is on the c-statistics and Brier scores.

**Table 5:** Model comparisons for health outcomes in all-age diabetes cohorts

	**Cohort 1**^**a **^**(*n *= 29,058)**				**Cohort 2 (*n *= 41,925)**			
**Model (df)**^b^	***c*-statistic**^c^**(95% CI)**	**∆*c***^d^**(%)**	**Brier score**	**LRT**^e^	***c*-statistic**^c^**(95% CI)**	**∆*c *(%)**	**Brier score**	**LRT**^e^
**AMI**								
Base (7)	0.66 (0.64, 0.68)	.	0.02	.	0.68 (0.66, 0.70)	.	0.02	.
# diagnoses (10)	0.67 (0.65, 0.69)	0.01 (1.37)	0.02	21.1^*^	0.68 (0.67, 0.71)^*^	<0.01 (1.47)	0.02	32.1^*^
Charlson (10)	0.67 (0.65, 0.69)	0.01 (1.52)	0.02	21.5^*^	0.69 (0.67, 0.71)^*^	0.01 (1.92)	0.02	37.3^*^
Elixhauser (35)	0.68 (0.66, 0.69)^*^	0.02 (2.43)	0.02	44.2^*^	0.70 (0.68, 0.72)^*^	0.02 (2.95)	0.02	62.5^*^
# drugs (10)	0.68 (0.66, 0.69)^*^	0.02 (2.43)	0.02	36.0^*^	0.68 (0.66, 0.70)	<0.01 (0.88)	0.02	24.1^*^
CDS (11)	0.67 (0.65, 0.69)^*^	0.01 (2.28)	0.02	32.8^*^	0.68 (0.66, 0.70)	<0.01 (0.29)	0.02	6.1
**Stroke**								
Base (7)	0.70 (0.68, 0.72)	.	0.02	.	0.71 (0.69, 0.72)	.	0.01	.
# diagnoses (10)	0.71 (0.70, 0.73)	0.01 (1.57)	0.02	37.8^*^	0.71 (0.69, 0.73)	<0.01 (0.85)	0.01	18.9^*^
Charlson (10)	0.72 (0.70, 0.73)	0.02 (1.85)	0.02	44.3^*^	0.71 (0.69, 0.73)	<0.01 (0.85)	0.01	17.2^*^
Elixhauser (35)	0.72 (0.70, 0.74)^*^	0.02 (2.56)	0.02	45.4^*^	0.71 (0.69, 0.73)	<0.01 (0.85)	0.01	20.6
# drugs (10)	0.71 (0.69, 0.73)	0.01 (0.85)	0.02	19.5^*^	0.71 (0.70, 0.73)	<0.01 (0.99)	0.01	24.2^*^
CDS (11)	0.71 (0.69, 0.73)	0.01 (1.28)	0.02	33.7^*^	0.71 (0.70, 0.73)	<0.01 (0.99)	0.01	21.2^*^
**LEA**								
Base (7)	0.65 (0.62, 0.68)	.	0.01	.	0.68 (0.65, 0.70)	.	0.01	.
# diagnoses (10)	0.71 (0.69, 0.74)^*^	0.06 (10.36)	0.01	77.4^*^	0.74 (0.72, 0.77)^*^	0.06 (9.75)	0.01	128.7^*^
Charlson (10)	0.74 (0.71, 0.77)^*^	0.09 (14.06)	0.01	128.4^*^	0.76 (0.74, 0.79)^*^	0.08 (12.70)	0.01	201.7^*^
Elixhauser (35)	0.78 (0.75, 0.81)^*^	0.13 (20.71)	0.01	305.6^*^	0.79 (0.76, 0.82)^*^	0.11 (16.69)	0.01	372.8^*^
# drugs (10)	0.70 (0.67, 0.72)^*^	0.05 (7.42)	0.01	53.0^*^	0.74 (0.72, 0.76)^*^	0.06 (9.31)	0.01	110.2^*^
CDS (11)	0.69 (0.66, 0.72)^*^	0.04 (6.34)	0.01	42.4^*^	0.72 (0.70, 0.75)^*^	0.04 (6.65)	0.01	74.5^*^
**ESRD**								
Base (7)	0.67 (0.63, 0.72)	.	<0.01	.	0.65 (0.61, 0.69)	.	<0.01	.
# diagnoses (10)	0.74 (0.70, 0.79)^*^	0.07 (10.73)	<0.01	49.0^*^	0.77 (0.73, 0.80)^*^	0.12 (18.52)	<0.01	128.0^*^
Charlson (10)	0.80 (0.76, 0.84)^*^	0.13 (19.08)	<0.01	138.6^*^	0.82 (0.79, 0.86)^*^	0.17 (27.01)	<0.01	276.6^*^
Elixhauser (35)	0.84 (0.80, 0.89)^*^	0.17 (25.78)	<0.01	328.6^*^	0.87 (0.84, 0.90)^*^	0.22 (34.41)	<0.01	555.1^*^
# drugs (10)	0.73 (0.69, 0.76)^*^	0.06 (8.20)	<0.01	36.6^*^	0.71 (0.68, 0.75)^*^	0.06 (9.88)	<0.01	50.2^*^
CDS (11)	0.75 (0.71, 0.79)^*^	0.08 (12.07)	<0.01	61.3^*^	0.72 (0.68, 0.76)^*^	0.07 (11.11)	<0.01	55.3^*^
**Hospitalization, any reason**								
Base (7)	0.63 (0.62, 0.64)	.	0.24	.	0.63 (0.62, 0.63)	.	0.24	.
# diagnoses (10)	0.70 (0.69, 0.71)^*^	0.07 (11.13)	0.22	2115.1^*^	0.70 (0.69, 0.70)^*^	0.07 (11.31)	0.22	3101.3^*^
Charlson (10)	0.68 (0.67, 0.69)^*^	0.05 (8.11)	0.23	1424.1^*^	0.67 (0.67, 0.68)^*^	0.04 (7.32)	0.23	1794.8^*^
Elixhauser (35)	0.69 (0.68, 0.69)^*^	0.06 (9.06)	0.22	1663.7^*^	0.68 (0.68, 0.69)^*^	0.05 (8.60)	0.23	2165.5^*^
# drugs (10)	0.66 (0.66, 0.67)^*^	0.03 (5.09)	0.23	823.1^*^	0.65 (0.65, 0.66)^*^	0.02 (3.98)	0.23	996.2^*^
CDS (11)	0.65 (0.64, 0.66)^*^	0.02 (3.50)	0.23	539.2^*^	0.64 (0.64, 0.65)^*^	0.01 (2.23)	0.24	503.0^*^
**Hospitalization, diabetes**								
Base (7)	0.60 (0.59, 0.61)	.	0.18	.	0.59 (0.59, 0.60)	.	0.15	.
# diagnoses (10)	0.65 (0.65, 0.66)^*^	0.05 (8.65)	0.18	889.9^*^	0.66 (0.65, 0.66)^*^	0.07 (10.83)	0.15	1251.0^*^
Charlson (10)	0.66 (0.65, 0.67)^*^	0.06 (9.65)	0.17	1194.1^*^	0.65 (0.64, 0.66)^*^	0.06 (9.98)	0.15	1141.8^*^
Elixhauser (35)	0.67 (0.66, 0.68)^*^	0.07 (11.65)	0.17	1554.3^*^	0.66 (0.66, 0.67)^*^	0.07 (12.35)	0.15	1715.9^*^
# drugs (10)	0.64 (0.63, 0.64)^*^	0.04 (5.66)	0.18	524.5^*^	0.63 (0.62, 0.63)^*^	0.04 (6.09)	0.15	633.9^*^
CDS (11)	0.63 (0.62, 0.63)^*^	0.03 (4.16)	0.18	365.1^*^	0.62 (0.61, 0.63)^*^	0.03 (4.57)	0.15	397.7^*^
**Death**								
Base (7)	0.77 (0.76, 0.78)	.	0.08	.	0.79 (0.78, 0.79)	.	0.07	.
# diagnoses (10)	0.79 (0.78, 0.80)^*^	0.02 (3.39)	0.08	499.4^*^	0.80 (0.80, 0.81)^*^	0.01 (2.16)	0.07	475.1^*^
Charlson (10)	0.82 (0.81, 0.82)^*^	0.05 (6.53)	0.07	1014.0^*^	0.82 (0.82, 0.83)^*^	0.03 (4.57)	0.07	1018.0^*^
Elixhauser (35)	0.83 (0.82, 0.83)^*^	0.06 (7.96)	0.07	1379.9^*^	0.84 (0.83, 0.84)^*^	0.05 (6.10)	0.06	1618.7^*^
# drugs (10)	0.79 (0.78, 0.80)^*^	0.02 (2.74)	0.08	429.1^*^	0.80 (0.79, 0.81)^*^	0.01 (1.78)	0.07	401.4^*^
CDS (11)	0.78 (0.77, 0.79)^*^	0.01 (1.96)	0.08	306.1^*^	0.79 (0.79, 0.80)^*^	<0.01 (0.89)	0.07	216.9^*^

^a^Cohort 1 was defined using administrative data from fiscal years 1996/97 to 1997/98 and Cohort 2 was defined using data from 1996/97 to 2001/02; ^b^Base model includes covariates of age, age^2^, sex, income quintile, geography, and recent diabetes diagnosis, and full models include these variables in addition to the specified comorbidity measure; ^c^A *c*-statistic with ^*^ is significantly different (p < .01) from the *c*-statistic for the base model, ^d^∆*c* = *c*-statistic for full model minus *c*-statistic for base model, ^e^A likelihood ratio test (LRT) with ^*^ is statistically significant at *α* = .01; df=degrees of freedom, *CDS* Chronic Disease Score, *AMI* Acute Myocardial Infarction, *LEA* Lower Extremity Amputation, *ESRD* End-Stage Renal Disease.

For AMI, the base models had *c*-statistics of 0.66 (95% CI: 0.64, 0.68) and 0.68 (95% CI: 0.66, 0.70) in Cohorts 1 and 2, respectively and both had a Brier score of 0.02, indicating poor predictive performance and low error. The addition of a comorbidity measure was associated with, at most, a 2.95% increase in the *c*-statistic. None of the full models had *c*-statistics that exceeded 0.70.

The base model for stroke in Cohort 1 had a *c*-statistic of 0.70 (95% CI: 0.68, 0.72) and a Brier score of 0.02, indicating good discrimination and low error. The improvement in the *c*-statistic was only statistically significant for the Elixhauser index (2.56%). The *c*-statistic for the base model in Cohort 2 was similar but the percentage change in this measure was not statistically significant for any of the full models.

For LEA, the *c*-statistic for the base model in Cohort 1 was below 0.70; each comorbidity measure resulted in a statistically significant increase in the *c*-statistic. The largest improvement was for the Elixhauser index (20.71%), followed by the Charlson index (14.06%). Both indices had low Brier scores (0.01). Similar results were found for Cohort 2, although the *c*-statistics were higher for the full models, and the change in the *c*-statistics was, overall, smaller than for Cohort 1.

Similar findings were observed for ESRD. In Cohort 1, there was excellent discrimination for the full model containing the Elixhauser index (*c* = 0.84; 95% CI: 0.80, 0.89). In Cohort 2, both the Elixhauser index (*c* = 0.87; 95% CI: 0.84, 0.90) and Charlson index (*c* = 0.82; 95% CI: 0.79, 0.86) resulted in full models with excellent predictive performance.

The base models for the two hospitalization outcomes had lower *c*-statistics and higher Brier scores than the base models for disease-specific outcomes. While all of the comorbidity measures resulted in statistically significant improvements in the *c*-statistic, none of the full models had values greater than 0.70. For hospitalization for any reason, the largest improvement was observed for the number of different diagnoses. For diabetes hospitalization, the largest improvement was observed for the Elixhauser index, but it was similar to the value for the number of diagnoses.

For death, the *c*-statistic of the base model for Cohort 1 was 0.77 (95% CI: 0.76, 0.78) and the Brier score was 0.08, indicating good discrimination and low prediction error. Results were similar for Cohort 2. All comorbidity measures were associated with statistically significant increases in the *c*-statistic. In Cohort 1, the largest increase was for the Elixhauser index (*c* = 0.83; 95% CI: 0.82, 0.83) followed by the Charlson index (*c* = 0.82; 95% CI: 0.81, 0.82). Similar results were found for Cohort 2, although the percentage change in the *c*-statistics were smaller than for Cohort 1.

The age-specific results are reported in Table 6. We conducted the analyses for death only, to limit the number of model comparisons and also because for some outcomes, age-specific models could not be fit to the data given the low numbers of health events. LRT statistics for all models were statistically significant, except for the model for number of drugs in the 20 to 44 age group in Cohort 1.

**Table 6:** Model comparisons for death in age-specific diabetes cohorts

	**Cohort 1**^**a **^**(*n *= 29,058)**				**Cohort 2 (*n *= 41,925)**			
**Model (df)**^b^	***c*-statistic**^c^**(95% CI)**	**∆*c***^d^**(%)**	**Brier score**	**LRT**^f^	***c*-statistic**^c^**(95% CI)**	**∆*c *(%)**	**Brier score**	**LRT**^f^
**20-44 years**								
Base (7)	0.65 (0.56, 0.74)	.	0.01	.	0.64 (0.57, 0.71)	.	0.01	.
# diagnoses (10)	0.74 (0.65, 0.83)	0.09 (13.02)	0.01	14.1^*^	0.75 (0.69, 0.81)^*^	0.11 (16.82)	0.01	43.1^*^
Charlson (10)	0.81 (0.74, 0.89)^*^	0.16 (24.20)	0.01	50.1^*^	0.80 (0.74, 0.86)^*^	0.16 (24.61)	0.01	87.9^*^
Elixhauser^e^	--	--	--	**--**	0.72 (0.66, 0.79)^*^	0.08 (12.77)	0.01	36.2^*^
# drugs (10)	0.72 (0.64, 0.80)	0.07 (10.26)	0.01	9.0	0.71 (0.65, 0.78)^*^	0.07 (11.06)	0.01	21.1^*^
CDS	--	--	--	**--**	0.70 (0.63, 0.77)	0.06 (9.35)	0.01	19.0^*^
**45-64 years**								
Base (7)	0.62 (0.59, 0.65)	.	0.04	.	0.62 (0.59, 0.64)	.	0.03	.
# diagnoses (10)	0.72 (0.69, 0.74)^*^	0.10 (15.81)	0.04	149.0^*^	0.69 (0.67, 0.72)^*^	0.07 (12.85)	0.03	149.0^*^
Charlson (10)	0.76 (0.74, 0.79)^*^	0.14 (22.74)	0.04	345.0^*^	0.74 (0.72, 0.76)^*^	0.12 (20.33)	0.03	368.2^*^
Elixhauser (35)	0.77 (0.74, 0.79)^*^	0.15 (23.71)	0.04	416.7^*^	0.75 (0.73, 0.77)^*^	0.13 (21.95)	0.03	416.7^*^
# drugs (10)	0.60 (0.66, 0.71)^*^	0.08 (10.97)	0.04	103.5^*^	0.68 (0.65, 0.70)^*^	0.06 (9.76)	0.03	103.5^*^
CDS (11)	0.66 (0.63, 0.69) ^*^	0.04 (6.29)	0.04	60.1^*^	0.65 (0.62, 0.68)^*^	0.03 (5.69)	0.03	60.1^*^
**65-74 years**								
Base (7)	0.60 (0.58, 0.62)	.	0.09	.	0.59 (0.57, 0.61)	.	0.08	.
# diagnoses (10)	0.68 (0.66, 0.70)^*^	0.08 (13.88)	0.09	186.8^*^	0.66 (0.64, 0.68)^*^	0.07 (12.05)	0.08	186.8^*^
Charlson (10)	0.73 (0.71, 0.75)^*^	0.13 (22.24)	0.08	407.0^*^	0.71 (0.69, 0.73)^*^	0.12 (20.03)	0.07	407.0^*^
Elixhauser (35)	0.75 (0.73, 0.76)^*^	0.15 (24.58)	0.08	548.8^*^	0.72 (0.70, 0.74)^*^	0.13 (22.24)	0.07	548.8^*^
# drugs (10)	0.67 (0.65, 0.69)^*^	0.07 (11.71)	0.09	131.1^*^	0.65 (0.63, 0.67)^*^	0.06 (9.68)	0.08	131.1^*^
CDS (11)	0.66 (0.64, 0.68)^*^	0.06 (9.70)	0.09	63.0^*^	0.62 (0.60, 0.64)^*^	0.03 (4.92)	0.08	63.0^*^
**75+ years**								
Base (7)	0.64 (0.62, 0.66)	.	0.16	.	0.67 (0.65, 0.68)	.	0.16	.
# diagnoses (10)	0.67 (0.66, 0.69)^*^	0.03 (4.84)	0.16	159.7^*^	0.69 (0.68, 0.70)^*^	0.02 (3.60)	0.16	159.7^*^
Charlson (10)	0.70 (0.68, 0.71)^*^	0.06 (8.91)	0.16	315.3^*^	0.71 (0.70, 0.72)^*^	0.04 (6.76)	0.15	318.0^*^
Elixhauser (35)	0.72 (0.70, 0.73)^*^	0.08 (12.34)	0.15	633.5^*^	0.74 (0.73, 0.75)^*^	0.07 (11.11)	0.15	633.5^*^
# drugs (10)	0.68 (0.66, 0.69)^*^	0.04 (5.47)	0.16	161.2^*^	0.69 (0.68, 0.70)^*^	0.02 (3.45)	0.16	161.2^*^
CDS (11)	0.67 (0.65, 0.68)^*^	0.03 (4.22)	0.16	95.1^*^	0.68 (0.67, 0.69)^*^	0.01 (1.95)	0.16	95.1^*^

^a^Cohort 1 was defined using data from fiscal years 1996/97 to 1997/98 and Cohort 2 was defined using data from 1996/97 to 2001/02; ^b^Base model includes age, age^2^, sex, income quintile, geography, and recent diabetes diagnosis, and full models include these variables in addition to the specified comorbidity measure; ^c^A *c*-statistic with ^*^ is significantly different (p < .01) from the *c*-statistic for the base model; ^d^∆*c* = *c*-statistic for full model minus *c*-statistic for base model; ^e^Some full models failed to converge due to the small number of deaths; ^f^A likelihood ratio test (LRT) with ^*^ is statistically significant at *α* = .01; df=degrees of freedom; *CDS* Chronic Disease Score.

For each age group, the base model *c*-statistic was consistently below 0.70. Brier scores were smallest for the youngest age group and largest for the oldest age group. For Cohort 1 in the youngest age group, only the Charlson index resulted in a significant increase in the *c*-statistic. In Cohort 2 in the youngest age group, the Charlson index, number of different diagnoses, Elixhauser index and number of different prescription drugs resulted in statistically significant increases in the *c*-statistic. The results for the other age groups were similar in the two cohorts. The addition of each comorbidity measure to the base model was associated with a statistically significant increase in the *c*-statistic. The Elixhauser index consistently resulted in the largest increase in the *c*-statistic, followed by the Charlson index.

## Conclusions

This study of comorbidity measures in population-based cohorts with diagnosed diabetes had the following key findings. First, there were substantial differences in the predictive performance of the base set of risk-adjustment variables selected for this study. Performance was lowest for hospitalization measures and highest for death and stroke. Improvements in model fit were often observed when a comorbidity measure was included in the model. However, for the health outcomes of AMI and stroke, there was limited utility associated with the inclusion of a comorbidity measure in the risk-adjustment model, based on model discrimination (i.e., *c*- statistic). For the other health outcomes, there was always a statistically significant improvement in the *c*-statistic for the full models. ESRD and death were the outcomes for which the comorbidity measures resulted in the greatest improvement in predictive performance. The model containing the Elixhauser index had the best predictive performance for all outcomes except for hospitalization for any reason, where number of diagnoses performed well. However, this was not always the case when age-specific cohorts were investigated. Similar changes in the *c*-statistics were observed for the diagnosis-based comorbidity measures regardless of whether the measures were based on ICD-9 codes only, or both ICD-9 and ICD-10 codes. The comorbidity measures based on prescription drugs had similar changes in the *c*-statistic values in the two cohorts to those observed using the diagnosis-based measures. Overall, however, comorbidity measures based on diagnosis codes performed better than comorbidity measures based on prescription drug codes.

The findings that the Charlson and Elixhauser indices performed well for predicting general measures of hospital utilization and mortality concurs with previous research [10,45,46]. However, this research has also shown that predictive performance of these comorbidity measures tends to be lower for healthcare utilization than for mortality, but still greater than when the predictive model is limited to socio-demographic variables and recency of diagnosis. Farley et al. [45] found that for predicting healthcare expenditures in the general population, simple count measures, such as counts of the number of diagnosis clusters, performed better than the Charlson and Elixhauser indices, which is consistent with most of the findings of the current study.

Schneeweiss et al. [47] observed that in a population of older adults, comorbidity measures based on medication codes had poorer performance than measures based on diagnosis codes. We also observed this for the age-specific analyses. The percentage change in the *c*-statistic for the full models containing the CDS and number of different drugs was larger in younger than in older age groups. The poor performance of measures based on prescription drug codes may arise because we focused on short-term outcomes and some drugs are used by individuals primarily for preventive therapy, as opposed to being used for treatment of chronic conditions. Finally, the addition of new drug classes to the marketplace since the CDS was originally developed may also contribute to its poorer predictive performance.

An interesting finding was that members of Cohort 1 had fewer comorbid conditions but were more likely to experience a health outcome compared to members of Cohort 2, who had a greater burden of comorbidity but were less likely to experience a health outcome. This observation of greater comorbidity could potentially be explained by the increase in the number of diagnostic fields from three in 1997/98 to 25 in 2001/02 in hospital administrative data. However, this does not appear to have affected the overall predictive performance of the comorbidity measures. The finding that predictive performance of comorbidity measures was not substantially different when diagnoses were based on ICD-9 only compared to when they were based on both ICD-9 and ICD-10 is consistent with previous research [12,13].

There are some limitations to this study. Comorbidity was defined using only a single year of data and was based on data from the year immediately prior to the outcome observation period. However, this methodology parallels one adopted in a similar study involving a general elderly population [10]. Moreover, previous research found that varying the time frame for measurement of comorbidity had a trivial effect on predictive performance [48,49]. The study cohorts were not independent; 80% of the individuals in Cohort 1 were also present in Cohort 2. It would have been preferable to examine predictive performance in independent cohorts defined over the same period of time, with different ICD coding systems being used in parallel, to avoid the potential confounding effects of cohort aging and changes in ICD coding on predictive performance. Sundarajan et al. [14] also noted the potential for temporal confounding in their investigation of changes in ICD coding. However, a study design that used independent cohorts was not possible to implement to evaluate the potential effects of the change in diagnosis coding. We observed that the prevalence of comorbid conditions was similar in both cohorts, with the exception of uncomplicated hypertension, suggesting little change in capture of major comorbidities with a change in diagnosis coding. Other comorbidity measures could have been included in the analysis. For example, an updated version of the CDS has been developed, [50] although Schneeweiss et al. [47] found that this revision did not result in improved predictive performance when compared to the original CDS in an elderly population. Another limitation is that some of the investigated outcomes were sparse in the cohorts, which can reduce the power of Delong’s [41] test for differences in discriminative performance of the models [51]. Finally, it is generally recognized that when working with administrative data, misclassification may arise due to inaccuracies in the assignment of diagnostic codes [32]. For example, rule-out diagnoses, which are used to indicate that an individual does not have a condition, may be incorrectly classified as comorbidities.

Major strengths of this study are the investigation of multiple outcome measures, several commonly-used general measures of comorbidity and measures based on both diagnosis and prescription drug codes. As well, we conducted age-specific analyses as well as analyses for all-ages cohorts to assess the generalizability of performance of comorbidity measures across the population. Using population-based data as opposed to data for a specific clinical cohort improves generalizability of the study results. Finally, our base model included a variety of variables that can be validly defined using administrative data and a broad range of potential risk variables.

In summary, our study suggests that the predictive performance of comorbidity measures based on administrative health data in population-based diabetes cohorts will vary with the outcome measure under investigation, although the Elixhauser index performed well overall. Predictive performance of all measures may not be equivalent for all age groups. At the same time, changes in the diagnosis coding system used in hospitalization data do not appear to affect predictive performance over time.

## Competing interests

The authors declare that they have no competing interests.

## Authors’ contributions

LML and JQ conducted the data extraction and carried out the statistical analyses. OF summarized the data. LML, JQ, and OF drafted the manuscript. LML, JQ, and GFT conceived the study and participated in its design. All authors read and approved the final manuscript.

## Pre-publication history

The pre-publication history for this paper can be accessed here:

http://www.biomedcentral.com/1472-6963/13/340/prepub
